# GDGT distribution in a stratified lake and implications for the application of TEX_86_ in paleoenvironmental reconstructions

**DOI:** 10.1038/srep34465

**Published:** 2016-10-03

**Authors:** Zhaohui Zhang, Rienk H. Smittenberg, Raymond S. Bradley

**Affiliations:** 1 Institute of Marine Chemistry and Environment, Ocean College, Zhejiang University, 1 Zheda Road, Zhoushan, 316021, China; 2 Climate System Research Center, Department of Geosciences, University of Massachusetts, 627 North Pleasant Street, Amherst, MA 01003-9354, USA; 3 Department of Geological Sciences, Stockholm University, Svante Arrhenius väg 8, SE-106 91 Stockholm, Sweden

## Abstract

We investigated the relationship between distributions of GDGTs, GDGT-based proxies and environmental factors in a stratified lake in northwestern Norway. More than 90% of isoGDGTs were produced at the bottom of the oxycline, indicating a predominance of ammonia-oxidizing Group I.1a of Thaumarchaeota, supported by high crenarchaeol/caldarchaeol ratios. Dissolved oxygen content, rather than temperature, exercised a primary control on TEX_86_ values. In spite of low BIT value in surface sediment, the reconstructed lake surface temperature was “cold” biased. MBT values in streams and lake surface water were significantly smaller than those in the catchment soil, suggesting *in situ* production of brGDGTs in streams. A rapid transition of MBT vs. temperature/pH relationships occurring at the bottom of oxycline indicated the differential production of various brGDGTs with D.O. and depths. Only within the oxycline were CBT-based pH values close to *in situ* pH. Our results confirm earlier studies calling for caution in applying TEX_86_ as a surface temperature proxy, or MBT and/or CBT for reconstructing pH, in anoxic or euxinic lakes, estuaries and ocean basins. We propose that caldarchaeol/crenarchaeol ratio, an indicator of contributions from methanogenic archaea, together with the BIT and TEX_86_ proxies, can help reconstruct past levels of stratification.

Members of the Thaumarchaeota (formerly Marine Crenarchaeota Group I)^1^ synthesize glycerol-dialklyl-glycerol-tetraethers (GDGTs) with 0–4 cyclopentane moieties as well as crenarchaeol, which contains a cyclohexane moiety in addition to four cyclopentane moieties^2^ (GDGTs I-V; see Supplementary Fig. S1). A molecular paleotemperature proxy, TEX_86_, was developed based on the relationship between relative abundance of cycloalkyl moieties in isoprenoid GDGTs (isoGDGTs)^3^ (GDGTs I-V; see Supplementary Fig. S1) and sea surface temperature (SST), and further calibrated based on a suite of ocean surface sediments^4^. The proxy has also been tested and applied in lakes to reconstruct past lake surface temperatures (LST)^5^,^6^,^7^.

Sediments from stratified basins and lakes such as the Cariaco Basin and the Mediterranean^8^,^9^ constitute excellent archives of climate and environmental changes because of their high sedimentation rates, quick response times, and good preservation of organic matter and biomarkers. TEX_86_-based reconstructions of SSTs of stratified basins are, however, often substantially different from reconstructions based on other proxies. For example, in the eastern Mediterranean Basin, TEX_86_-based SSTs reconstructed from sapropels (indicating anoxic conditions) were 15–17 °C, significantly lower than UK_37_-based SSTs, ca. 25 °C^10^. TEX_86_- reconstructed temperatures from sediment traps in the Santa Barbara Basin were also substantially lower than SST^11^. Clearly, further understanding of factors other than temperature influencing the distribution of isoGDGTs is essential before the proxy can be applied successfully in dysoxic or anoxic settings. A first step towards such understanding is to map out the production of isoGDGTs *in situ* in a water column as a response to parameters such as temperature, pH, redox conditions, etc.

A general feature of stratified basins is the depletion of dissolved oxygen (D.O.) and transformation of nitrogen species with depth in the water columns. A recent investigation of two marine ammonia-oxidizing Thaumarchaeota cultures demonstrated that oxygen concentration was at least as important as temperature in controlling TEX_86_ values: both higher growth temperatures and reduced levels of D.O. resulted in higher TEX_86_ values^12^. Elevated TEX_86_ values were mainly the result of the relative increase in GDGT-II under low O_2_ concentrations as a lipid biosynthetic response^12^. NH_4_^+^ limitation, which is metabolically similar to O_2_ limitation, was also shown to influence TEX_86_ in the cultures of thaumarchaeon *Nitrosopumilus maritimus*^13^. It would be reasonable to assume similar responses under natural conditions of low D.O. levels in stratified basins in addition to changes in dominant archaeal species.

Distributions of a second main group of GDGTs, the branched GDGTs (brGDGTs) (GDGT VI-VIII; See Supplementary Fig. S1) initially found in soils, have been shown to correlate with temperature and pH^14^,^15^,^16^. Over the last decade the methylation index MBT and cyclisation index CBT have been developed to reconstruct temperature and pH via a variety of calibrations^16^,^17^,^18^,^19^,^20^. The applications of the combined MBT/CBT proxy in lake/marine sediments have been based on the assumption that brGDGTs are produced only in catchment soils. However, recently the CBT index was also found to be related to lake conductivity and alkalinity^21^,^22^, and there is more recent evidence of *in situ* production of brGDGTs^23^,^24^. This may explain the often unsuccessful application of the MBT/CBT proxy in marine environment^25^. More knowledge about other parameters dictating brGDGT distributions could alleviate this problem, and offer the opportunity of even finding new brGDGT-based proxies.

In this study, we examined the distributions of core GDGTs in the water column and sediment of a density-stratified coastal lake in the Lofoten region, Norway (Fig. 1) to understand the factors controlling the contribution of various GDGTs to the sediments. We measured depth profiles of isoprenoid and branched GDGTs along with those of salinity, temperature, dissolved oxygen and pH *in situ*, which enabled us to evaluate non-temperature biochemical factors controlling the production, distributions of GDGTs, and the GDGT-based indices.

## Results

The water column in the lake, measured in September 2007, was stratified, as indicated by combined thermo-, halo-, pycno- and chemo-clines (Fig. 2A–D; Table 1). Salinity appeared to be the driving factor of density stratification below 6.6 m, which was exacerbated by the strong thermocline between 15–19 m (Fig. 2C). The oxycline started at a shallower depth than the thermocline, but they all reached a minimum at 19.5 m (Fig. 2A,D). A first abrupt decline in pH occurred at 6.6 m, and a second happened within the oxycline, with the minimum at the oxycline bottom (Fig. 2E; Table 1). Nitrate was below the detection limit (<5 μg l^−1^) in the surface zone, increased rapidly below the photic zone, and reached the maximum at the base of oxycline, and then decreased substantially (Fig. 2G; Table 1). Ammonium was not detectable above the oxycline bottom, but its concentrations were extremely large below the oxycline (Fig. 2H; Table 1).

**Figure 1:**
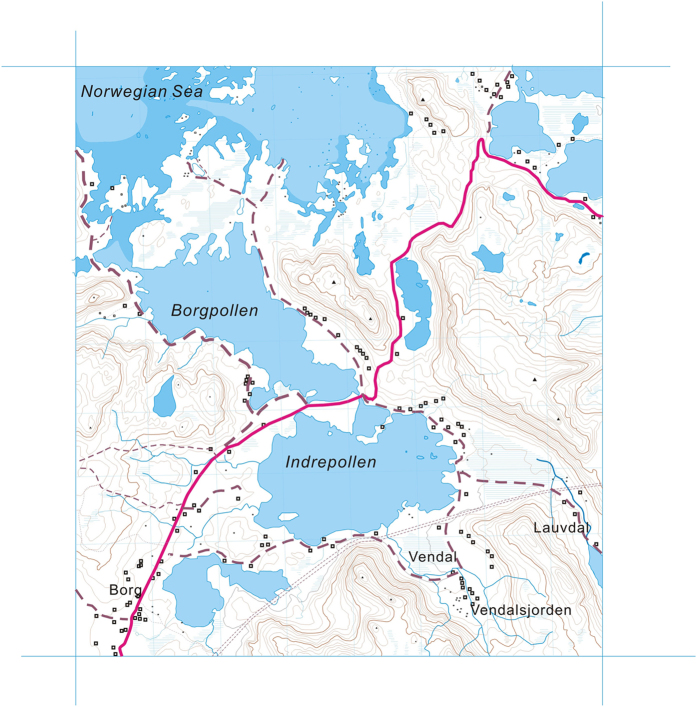
Map showing Lofoten Islands, the site of Indrepollen Lake and its connection to Borgpollen. Map was produced using Corel Draw X7 (www.coreldraw.com) and edited using Adobe Photoshop CS 2 (version 9.0).

**Figure 2:**
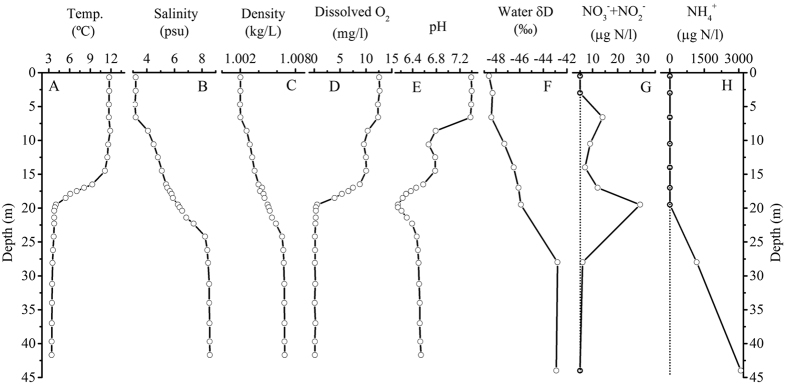
Measured physical parameters in the water column of Lake Indrepollen: (**A**) Temperature; (**B**) Salinity; (**C**) Density calculated from temperature and salinity using the equation^55^; (**D**) Dissolved oxygen content (D.O.); (**E**) pH; (**F**) δD values of water; (**G**) NO_3_^−^ + NO_2_^−^ concentration (μg N/l); (**H**) NH_4_^+^ concentration (μg N/l).

The range of hydrogen isotopic (δD) values of the water (−48.6 to −42.9‰, Fig. 2F), combined with the salinity values, indicates that seawater seepage from the adjacent lake, Borgpollen (which was connected to the sea by a shallow inlet) contributed approximately 20% to the deeper portion of the lake (Fig. 1). Although the water seepage promoted lake stratification, it appeared to be too small to influence the broad lake water chemistry which was driven mainly by local, faster occurring biogeochemical processes, as evidenced by rapid oxygen depletion with depth.

All concentrations of GDGTs reported here refer to core GDGTs as we did not perform hydrolysis, which means any intact polar GDGTs were outside the analytical window. Sources of core GDGTs include *in situ* hydrolysis of intact GDGTs after cell death when hydrolysis carried out by bacterial enzymes removes the polar head group of the intact GDGTs and yields core GDGTs. Hydrolyzing intact GDGTs in the polar fractions could potentially release extra core GDGTs^26^, although concentrations of intact GDGTs have previously been found negligible in the marine water column^27^,^28^,^29^. While the measured chemical parameters reflect snapshots of ongoing biogeochemical processes, core GDGTs might represent a time-integrated signal. On the other hand, however, the water column likely provides an environment for rapid hydrolysis of intact GDGTs into core GDGTs.

**Figure 3:**
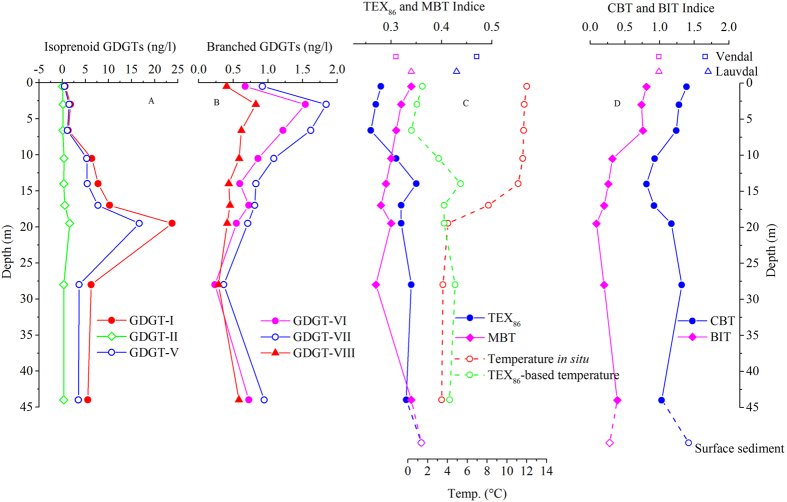
Distributions of individual isoprenoid and branched tetraether and GDGT-based proxies in the water column of Lake Indrepollen: (**A**) Concentrations of isoprenoid GDGTs; (**B**) Concentrations of branched GDGTs; (**C**) TEX_86_ and MBT indices, and the comparisons between TEX_86_–based temperature and *in situ* measurement; (**D**) CBT and BIT indices. Also shown are those parameters in the stream water and surface sediment.

Total isoGDGT concentrations increased rapidly with depth, and reached a maximum at the bottom of the oxycline (19.5 m), below which abundance of GDGTs decreased yet remained significantly higher than at the surface (Fig. 3A; Table S1). However, brGDGTs were highest in the upper water column and decreased with depth (Fig. 3B). The sample near the lake bottom had higher concentrations, likely resulting from resuspension from the sediment. As a consequence, the highest branched-over-isoprenoid tetraether (BIT) index^14^ value in the water column occurred at the lake surface (Fig. 3D) reflecting the predominant influence of brGDGTs from streams entering the lake through adjacent peat bogs (Tables 1 and S1).

All the isoprenoid GDGTs showed the same trend in the water column, with the predominance of caldarchaeol (GDGT-I), which was 10 times more abundant than GDGT-II, the next most abundant (Table S1). Concentrations of caldarchaeol were about 0.6 to 1.8 ng/l in the upper 6.6 m, but increased to 6.4 ng/l at 10.5 m, then increased dramatically to 23.7 ng/l at the oxycline bottom (19.5 m), and decreased to 6 ng/l below the oxycline (Table S1). Crenarchaeol (GDGT-V) and other isoGDGTs showed the same trends. In the catchment soil and peat, GDGTs-IV, -V and -V’ were below the detection limit, but GDGTs-I, -II and -III were present in very high concentrations (Table S1). In particular, GDGT-I at 1 m deep in the Lauvdal peat reached 2631 μg/g, indicating water-saturated and anoxic lower layers favored the production of caldarchaeol^30^. GDGT-V’ concentrations were also below the detection limit in both Lauvdal and Vendal streams (Table S1), but the concentrations of other isoGDGTs were similar to the lake surface water, suggesting the source of major input.

All the branched GDGTs, except GDGT-VIc and GDGT-VIIc, were present in the lake water column. GDGT-VII, the most abundant species, increased from 0.92 ng l^−1^ at the surface to a maximum of 1.84 ng l^−1^ at 3 m, then gradually decreased through the water column to reach a minimum of 0.36 ng l^−1^ at 28 m (Table 1; Fig. 3B). Their distributions were distinct from those of isoprenoid GDGTs in the water column. Branched GDGTs in the nearby soil and peat bogs were dominated by GDGT-VI and GDGT-VII. The total concentrations of brGDGTs in the surface peat was 17.41 μg g^−1^ but reached 61.83 μg g^−1^ at 1 m depth, substantially higher than those in soils (Table S1). The abundance of brGDGTs in Vendal stream water was twice as high as that in the Lauvdal stream (Fig. 1) and 8 times higher than that in lake surface water (Table S1).

## Discussion

The Indrepollen water column showed a classical profile of low NO_3_^−^ concentrations in the surface and abundant NH_4_^+^ below the oxycline, similar to those of other stratified water bodies^31^,^32^. A first nitrate maximum occurred at 6.6 m as a result of organic matter decomposing below the photic zone utilizing D.O., and the concentration gradually decreased with depth (Fig. 2D,G; Table 1). However, the trend of nitrate decline was reversed in the 17–19.5 m interval (Fig. 2G) while NH_4_^+^ was still negligible (Fig. 2H), hinting that at least part of the nitrate increment could be due to nitrification, which is evidenced by the sharp decline of D.O. (Fig. 2D). The co-occurrence of maximum production of isoGDGTs at the oxycline bottom and the rapid transition of nitrogen speicies from NO_3_^−^ to NH_4_^+^ (Figs 2G,H and 3A) indicates that ammonia-oxidizing (nitrifying) Group I.1a of Thaumarchaeota could be the major producers of isoGDGTs in this lake^33^. The sharp decline in the abundance of isoGDGTs below the oxycline suggests that such Thaumarchaeota could not survive without D.O.

The high BIT index (>0.7) in the upper water column indicates high terrestrial input, which typically leads to erroneous TEX_86_-based temperature estimates^34^,^35^. If the lake-based TEX_86_ calibration of Powers *et al.*^6^ is used, the TEX_86_ value at the surface (0.28) would correspond to approx. 1.5 °C, which is far apart from the temperature measured *in situ* (0–6.6 m, 12 °C) (Fig. 3C). The discrepancies between TEX_86_-based temperatures and *in situ* measurements decreased in the water column with the decline of D.O., and diminished within/below the oxycline/thermocline where BIT declined to less than 0.20 (Fig. 3C,D).

The surface sediment had a TEX_86_ value of 0.36 and a BIT index value of 0.28. The reconstructed temperature corresponds very well to the annual mean temperature observed around the oxycline, but was substantially lower than lake surface temperature. This discrepancy must be due to the fact that about 78% of the crenarchaeol in the Indrepollen water column was produced within the oxycline (between 10 and 19.5 m), and especially near the oxic/anoxic boundary. This estimate is based on summing up our GDGT concentration profile (Table S1), although it is difficult to accurately calculate the GDGT flux in different depths to sediment since efficiency/export mechanisms might vary with water depths. In general, most TEX_86_ ratio-based temperature reconstructions in paleotemperature studies assume that GDGT core lipids in lake or marine sediments derive quantitatively from exported biomass of surface-derived, planktonic, ammonia- oxidizing, autotrophic Thaumarchaeota^36^. However, our findings indicate that there is not always the predominance of isoGDGT production in the surface, particularly in water columns with low oxygen or depletion of oxygen at greater depth. This conclusion is supported by previously observed discrepancies in stratified environments. For example, in the eastern Mediterranean Basin and euxinic Black Sea, the TEX_86_-based SSTs were all significantly lower than UK_37_-based SSTs^10^,^37^. TEX_86_- reconstructed temperatures from sediment traps in the Santa Barbara Basin were substantially lower than SSTs, and the difference was attributed to the hypothesis that TEX_86_ in the SBB predominantly recorded subsurface temperatures (>100 m)^11^. These studies and our results suggest that Group I.1a of Thaumarchaeota thrive at the deeper and colder thermoclines/chemoclines in stratified water columns, resulting in a significant contribution of isoGDGTs from deeper water layers^38^. Microbial ecology studies also showed that the relative abundances of Thaumarchaeota increased in abundance at the oxycline^39^,^40^,^41^, and that archaeal ammonia monooxygenase transcript abundance increased in oxygen minimum zones of marine water columns^42^,^43^,^44^. Maximum levels of crenarchaeol were also detected within the oxygen minimum zone in the Arabian Sea^45^. A recent study revealed that sub- and anoxic layers of meromictic saline Lake Faro (Messina, Italy) were primarily inhabited by the organisms related to the clusters of Marine Group I.1a of Thaumarchaeota frequently recovered from oxygen-depleted marine ecosystems^46^. Such predominant habitation of Group I.1a of Thaumarchaeota at the oxycline bottom determined the distributions of GDGTs in the water columns and TEX_86_ signal in the surface sediments.

**Figure 4:**
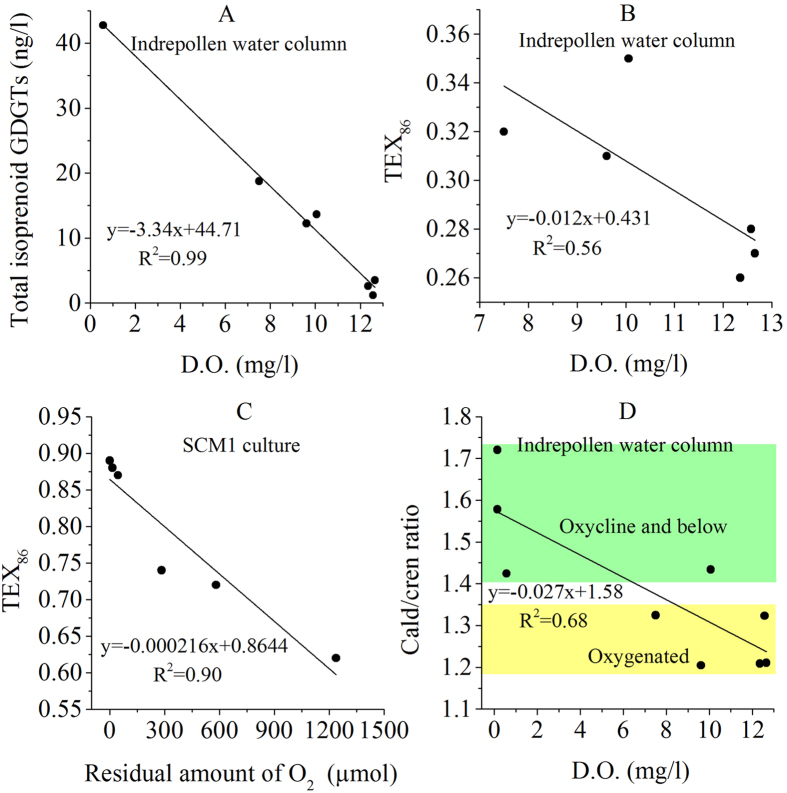
X-Y plots displaying the relationships between total isoprenoid concentration, TEX_86_ and caldarchaeol/crenarchaeol ratio vs. dissolved oxygen in the water column of Lake Indrepollen. (**A**) total isoprenoid concentration vs. D.O.; (**B**) TEX_86_ vs. D.O.; (**C**) TEX_86_ vs. residual amount of O_2_ in ammonia-oxidizing archaea SCM1 culture calculated from the reported data^12^; (**D**) caldarchaeol/crenarchaeol ratio vs. D.O.

The concentration of total isoGDGTs increased with the decline of D.O. until depletion was reached at the bottom of oxycline (19.5 m), forming a very strongly negative relationship (R^2^ = 0.99; Fig. 4A). This indicates the predominant influence of D.O. on production of isoGDGTs. In addition, GDGT-II increased with depth in a smaller amplitude than GDGT-III and GDGT-IV in response to declining D.O., resulting in increasing TEX_86_ values in the top 17 m (TEX_86_ = −0.013 × D.O. + 0.44; R^2^ = 0.56; Fig. 4B). Because of this, TEX_86_ values and temperatures *in situ* became negatively related (TEX_86_ = −0.005 × Temp + 0.35; R^2^ = 0.34), in the opposite direction of that expected from the calibrations based on surface sediments^3^,^4^. As a result, TEX_86_ and *in situ* water column temperatures were not related.

Recent studies highlighted environmental factors other than temperature influencing TEX_86_ in Thaumarchaeota^47^,^48^ in addition to the role of community composition^49^. Cultures of two marine ammonia-oxidizing archaea demonstrated that oxygen concentration played a role at least as important as temperature in controlling TEX_86_ values^12^. Using their data^12^, we calculated the relationship between the residual amount of O_2_ (μmol) and TEX_86_ values in the cultures of strain *Nitrosopumilus maritimus* SCM1 and got a nearly identical form to ours: TEX_86_ = −0.0002 × residual O_2_ + 0.86 (R^2^ = 0.78; Fig. 4C). Our results further suggest that if D.O. in a water column experiences a substantial change, it would play the predominant role over temperature in controlling TEX_86_ values. Furthermore, given the fact that all cultivated Thaumarchaeota are ammonia oxidizers, the most viable explanation for the observed negative relationship with D.O. is that they thrive best at the redox boundary where both NH_4_^+^ and D.O. were in their minima, which is supported by microbial genetic evidence^42^,^50^. In fact, Thaumarcheota may well play an active role in defining this redox boundary.

It is possible that a physiological response to low O_2_^12^ might be related to energy stress and a corresponding decrease in ammonia oxidation rates observed in the chemostatic culture of thaumarchaeon *Nitrosopumilus maritimus* SCM1^48^. On the other hand, the observed pattern could also be due to differential Thaumarchaeal TEX_86_-temperature responses between different species^12^,^48^, which adapted themselves in the depths with substantially different D.O. concentrations. As a result, the decline of D.O. in the water column might define the redox boundary, TEX_86_ values and distributions of archaea species.

Caldarchaeol (GDGT-I) has been reported to occur in both thermophilic Crenarchaeota and Euryarchaeota^51^, mesophilic Group I Thaumarchaeota^2^, as well as in methanogenic and anaerobic methane-oxidizing Euryarchaeota that mediate the anaerobic oxidation of methane^52^. Caldarchaeol was the most dominant GDGT in every sample (See Supplementary Table S1), which appeared to be a characteristic of freshwater and estuarine environments^27^. This was especially apparent in the Lauvdal peat bog, which only contained GDGT-I, II and III, and was lacking crenarchaeol, suggesting that methanogenic Euryarchaeota were dominant in the lower anoxic layer of peat bogs^30^, while Thaumarchaeota (crenarchaeol producers^2^,^53^) were absent. Yet, the occurrence of crenarchaeol in the fresh water streams indicates the presence of Thaumarchaeota where D.O. was abundant.

Since both crenarchaeol and caldarchaeol can be derived from Group I Thaumarchaeota, whereas methanogenic Euryarchaeota synthesize predominantly caldarchaeol but no crenarchaeol, the ratio of caldarchaeol/crenarchaeol (cald/cren ratio) can be used to indicate whether a major source of GDGTs in sediments is from methanogenic or nonmethanogenic Euryarchaeota^7^. In the Indrepollen water column, the cald/cren ratios were negatively related to D.O. in the water column: cald/cren = −0.0267 ×_ _D.O. + 0.576 (R^2^ = 0.68; Fig. 4D), providing clear evidence that depletion of D.O favored methanogenic Euryarchaeota and enhanced the production of caldarchaeol. Since the cald/cren ratio increased with the decline of D.O. in the Indrepollen oxycline (Table 1), it is very likely that methanogenic Archaea increased while Thaumarchaeota decreased in relative abundance within and below the oxycline, demonstrating that this ratio can serve as a proxy for the relative input of methanogenic Euryarchaeota vs. aquatic Thaumarchaeota. The cald/cren ratios were 1.2 to 1.3 in the top 10.5 m of the Indrepollen water column, and increased rapidly to 1.4~1.7 within/below the oxycline/thermocline (Fig. 4D). A ratio of 1.3~1.4 can be defined as the boundary (Fig. 4D), substantially less than 2 suggested previously^7^. Meanwhile, the BIT index changed from >0.7 in the upper water column to <0.26 in the deep portion, with a minimum of 0.09 at the bottom of oxycline. On the other hand, TEX_86_ values were <0.3 above the oxycline but >0.3 within/below it. A combination of the cald/cren ratio with BIT and TEX_86_, measured on a sediment record, may allow reconstruction of the depth and intensity of stratification, with lower cald/cren and TEX_86_ values but high BIT index indicative of a weak and deep (and cold) oxycline, and higher cald/cren ratios and TEX_86_ values but low BIT index suggesting the opposite situation. In addition, the relationship between D.O. and TEX_86_ as described above, if found valid also in other stratified environments, may provide a tool to reconstruct past levels of D.O.

The MBT indice in the water column showed a clearly declining trend with depth (in the top 17 m) before reaching the oxycline bottom (Fig. 3C), closely tracking decreasing temperature and lower pH *in situ*, and occurring in the same plane: MBT = 0.0362 × pH + 0.0032 × T + 0.0180. This relationship changed dramatically at the bottom of the oxycline, indicating the components of the methylation index can be produced at different ratios with depth within the water column. MBT values in the streams were close to lake surface water, but significantly smaller than those in the catchment soil and peat (Table S1), suggesting that *in situ* production of brGDGTs already occurred in streams^23^.

CBT values of around 1.4 in the soil and peat lead to an estimated pH value of around 5, which is reasonable for rain-fed peat systems, as CBT is generally considered to be controlled by soil pH^16^. However, the CBT value in lake surface water was 1.4, correponding to a pH of 5.1, significantly less than measured pH (7.4). Indeed, CBT and pH above the oxycline were positively related: CBT = 0.52 × pH-2.53 (R^2^ = 0.90), in contrast to the observations in soils^16^, reflecting the relationship between *in situ* pH and exogenous CBT signals. Within the oxycline, the relationship became similar to that in soils: CBT = 4.65−0.57 × pH (R^2^ = 0.95). In addition, only within the oxycline were CBT-based pHs close to pH measurements *in situ*, indicating the dominance of *in situ* production of brGDGTs in this zone, which resulted in the reverse of CBT variations in the water column (Fig. 3D).

MBT and CBT values in the surface sediment were close to those in the surface water (Table S1). However, if we sum up all branched GDGTs in the water column, the amount within the oxycline was about half the amount in the top 10 m (Table S1). Such a paradox suggests that brGDGTs produced in the oxycline were exported to the sediment much less efficiently.

The trends observed in the TEX_86_, MBT and CBT indices clearly indicate that they were affected by *in situ* environmental signals within the streams and water column, and we conclude that not only the isoprenoid GDGTs, but also the branched GDGTs were being produced and/or transformed within the streams and lake, as evidenced by the transition of MBT and CBT above and within/below the oxycline.

In spite of low BIT value in the lake surface sediment, the reconstructed lake surface temperature was substantially lower than measurements *in situ*, making a paleotemperature estimate “cold” biased (Fig. 3C; Table 1). Our results are opposite to recent observations of “TEX_86_ warming” due to oxygen depletion in the cultures, ocean and lakes^13^,^27^,^47^,^48^, but support previous findings of “cold temperature biases” in anoxic basins^10^,^11^. In Lake Indrepollen, there was a warming trend of TEX_86_ values in the water column coinciding with O_2_ depletion, but such trend only lasted till 14 m, the beginning of the sharp oxycline (Fig. 3C). Within the sharp decline of D.O. from 14 m to 19.5 m deep, TEX_86_ actually decreased. On the other hand, GDGTs were predominantly produced in and exported from the oxic/anoxic interface so that surface sediment TEX_86_ value was almost identical to that at the bottom of oxycline (Table 1). In fact the gap between TEX_86_ reconstructed temperature and *in situ* measurement was largest at the lake surface, but decreased with depth (and deline of D.O.) (Fig. 3C). Such a gap diminished at the bottom of the oxycline, indicating TEX_86_ *in situ* recorded the mean annual temperature of the oxycline, which was also reflected in the surface sediment. Apparently, there is more than one factor influcing the distribution of TEX_86_ in the water column. Change of community compositions with best adaption to different D.O. levels might also play an important role in addition to water temperature and physiological responses to declining D.O. Recognition of such discrepancies is of essential importance in reconstructing paleoclimate signals from stratified lakes, estuaries and ocean basins.

## Conclusions

Large amounts of crenarchaeol and other isoprenoid GDGTs existed right at the bottom of the oxycline, suggesting an active nitrogen cycle and a major contribution of Group I.1a of Thaumarchaeota within the oxycline. Dissolved oxygen exerted a predominant influence on the production of isoprenoid GDGTs, which increased in response to declining D.O. TEX_86_ values increased with the decline of D.O. and temperature *in situ* in the water column, which is opposite to conventional calibrations, and indicates that D.O. has a primary role in controlling TEX_86_ values, which are most likely unrelated to temperature.

As a result, the reconstructed lake surface temperature based on surface sediment TEX_86_ is substantially lower than measurement *in situ* in the surface, in spite of a low BIT value, in agreement with previous findings. TEX_86_ in the surface sediment did not reflect surface temperature, but recorded the mean annual temperature of the oxycline, making a paleotemperature application “cold” biased.

The caldarchaeol/crenarchaeol ratio was closely related to D.O., providing a potential tool to reconstruct past levels of D.O. in paleoenvironments. The larger caldarchaeol/crenarchaeol ratio within and below the oxycline (>1.4) than that in the upper water column (<1.4) indicated increased contribution of methanogenic archaea in the anoxic bottom waters. Temperatures reconstructed from TEX_86_ values in the surface water are substantially lower than measurements *in situ*. Only within/below the oxycline where BIT<0.2, was the discrepancy insignificant.

*In situ* production of brGDGTs occurred not only in streams as evidenced by similar MBT values in the streams and lake surface water (but significantly smaller than those in the catchment soil and peat), but also in the water column, in particular, the bottom of the oxycline. The relationship between MBT indices and temperature/pH changed dramatically above and below the interface, indicating the components of the methylation index could be produced at different ratios at different depths. A decreasing trend in CBT values with depth was found above the oxycline, positively related to pH and D.O. while the trend reversed below the oxycline. The CBT-based pH values were close to *in situ* pH only within the oxycline.

The trends observed in the TEX_86_, MBT and CBT indices clearly indicate that not only the isoprenoid GDGTs, but also the brGDGTs were produced within the streams and lake. Caution must be used when applying TEX_86_ for reconstructing surface water temperatures, or applying MBT and/or CBT for reconstructing pH in anoxic or euxinic lakes, estuaries and ocean basins. The combination of the caldarchaeol/crenarchaeol ratio and BIT and TEX_86_ proxies could potentially be used to aid in the reconstruction of past levels of stratification.

## Methods

### Study site

The lake Indrepollen is located in the Loften-Vesterålen archipelago (67–70°N), a chain of mountainous islands extending from the northeast to the southwest, from mainland Norway into the Norwegian Sea (Fig. 1). This once dynamic glacial region is characterized by many lakes, often in deeply eroded cirques (tarns), many of which are close to sea level. Indrepollen (68°44.444′N, 13°49.440′E) is situated on the island of Vestvågøy, part of the Lofoten archipelago and is a large lake-estuary system with multiple sedimentary basins^54^ (Fig. 1).

Indrepollen is currently very close to sea level (~ +1 m) and has a density-stratified water column. The main streams flowing into this lake are Lauvdal and Vendal (Fig. 1). The maximum water depth in the lake was 44 m in September, 2007. Indrepollen drained out through a very narrow connection (5 m wide, <0.5 m deep) to an adjacent low-lying lake, Borgpollen, which was in turn connected by a shallow channel to the Norwegian Sea (Fig. 1). During high tides, brackish waters from Borgpollen enter Indrepollen, reversing the normal flow regime through the outlet.

### Water column physical and chemical parameter measurements

Physical parameters including temperature, dissolved oxygen, pH and salinity were measured using a Hydrolab probe (MS5) at different depths in the water column. Densities were calculated from salinity and temperature^55^.

Data-logging thermistors were also deployed throughout the water column and recorded temperatures from September 2007 through August 2008. Nine thermistors spaced 5 m apart in the epilimnion and 10 m apart in the hypolimnion were suspended from a buoy anchored at the deepest location, and recorded temperature every 4 hours.

### Sample collection

Water sampling took place in September 2007 in the depocenter, about 1 km away from the brackish water seepage from Borgpollen (Fig. 1). Based on the temperature and D.O. profiles (Fig. 2A,D), water from a total of 9 selected depths (0.5, 3.0, 6.6, 10.6, 14.0, 17.0, 19.5, 28.0 and 44.0 m) was pumped *in situ* and collected in pre-cleaned plastic containers (100–125 liters). The shallowest one (0.5 m) was near the surface and the deepest one (44 m) was slightly above the bottom. A small aliquot was sealed in a 4-ml vial for water δD analysis, and another 500 ml aliquot was sealed in a brown bottle for nitrate/ammonia analyses. Those water samples were kept frozen until analyzed. The rest of the water was immediately filtered over pre-combusted 293 mm internal diameter GF/F filter (pore size 0.7 μm, Whatman).

Water from two streams flowing into the lake, Vendal (68°14.098′N, 13°51.368′E) and Lauvdal (68°14.343′N, 13°54.021′E) (Fig. 1), were filtered using the same protocol. All the filters were kept frozen at −80 °C until analyzed.

Three types of soil and peat samples in the catchment near the lake were collected and analyzed: one regular soil sample, two peat bog samples at different depths (surface and 1 m deep). Samples were kept frozen at −80 °C until analyzed.

A 1 m long water-mud interface sediment core was retrieved and surface sediment was used for this study.

### Water isotope analysis and nitrate/ammonia analyses

A total of 9 water samples from the different depths in the lake (0.5, 3.0, 6.6, 10.6, 14.0, 17.0, 19.5, 28.0 and 44.0 m) were analyzed for hydrogen isotope ratios at Dartmouth College^56^,^57^.

The above 9 water samples from the lake, and 2 water samples from the Vendal and Lauvdal streams were analyzed for NO_3_^−^ and NH_4_^+^ at the University of New Hampshire on a discrete colorimetric autoanalyzer (Westco Scientific, Smartchem 200), using methods based on EPA 353.2 (automated Cd-Cu reduction) and EPA 350.1 (automated phenate), respectively.

### Lipid extraction and GDGT analysis

GF/F filters of 9 lake waters and two stream waters were freeze-dried, cut into 0.5 × 0.5 cm pieces, and extracted on a Dionex ASE-200 pressurized fluid extractor with dichloromethane (DCM) and methanol (MeOH) (9:1) at 1200 psi and 100 °C^56^,^57^.

Three soil and peat samples and lake surface sediment were freeze dried and then extracted the same way. Total lipid extracts were further separated into apolar and polar fractions on column chromatography using activated Al_2_O_3_ as the stationary phase. Hexane:DCM (9:1 v/v) eluted the apolar fraction while the polar fractions containing the GDGTs were subsequently eluted by DCM-MeOH (1:1 v/v). After evaporation of the solvents the polar fractions were redissolved in a mixture of hexane:isopropanol (99:1 v/v, HPLC-grade) and filtered through an 0.45 μm PTFE filter (Alltech) prior to analysis. Prior to GDGT analysis 5 ng of a synthesized C_46_ GDGT standard was added to each sample as a quantification standard^58^.

No hydrolyses were performed so that intact polar-GDGTs were not analyzed.

The prepared samples were analyzed at the Geological Institute of the ETH Zurich using a Thermo Surveyor HPLC system interfaced via atmospheric pressure ionization to an LCQ Fleet ion trap Mass Spectrometer, equipped with a PAL LC autosampler and Xcalibur software. HPLC separation was performed using a normal phase Alltech Prevail Cyano column (150 mm × 2.1 mm; 3 μm) maintained at 30 °C. The flow rate of the hexane:isopropanol (IPA) (99:1) eluent was 0.3 ml min^−1^, isocratically for the first 5 min, thereafter with a linear gradient to 2% IPA in 30 min. The column was cleaned every six samples using 30% IPA in hexane, and re-equilibrated. Injection volume of the samples was 20–50 μl. Scanning was performed over the *m/z* ranges 740–746, 1016–1054 and 1280–1318. Quantification of the compounds was achieved using peak areas of the protonated molecular ions [M + H]^+^ and [M + 1 + H]^+^ in relation to those of the internal standard. A relative response factor of 4.0 was used to correct for differences in the response of the internal standard and the natural GDGT’s^58^, as an instrument-specific response factor could not yet be determined.

As the internal standard was not added in the lake surface sediment extraction, the abundances of individual GDGTs were not available. TEX_86_, MBT and CBT indices were calculated based on literature^3^,^16^. Measurement uncertainties for GDGT-based proxies are about 0.005.

## Additional Information

**How to cite this article**: Zhang, Z. *et al.* GDGT distribution in a stratified lake and implications for the application of TEX_86_ in paleoenvironmental reconstructions. *Sci. Rep.* **6**, 34465; doi: 10.1038/srep34465 (2016).

## Supplementary Material

Supplementary Information

## Figures and Tables

**Table 1:** Physical parameters and GDGT-based indices in water column, stream water and surrounding soils in Lake Indrepollen.

	Measured parameters						Calculated density	GDGT-based indice				
	Temp (°C)	D.O. (mg/l)	pH	δD (‰)	NO_3_^−^ + NO_2_^−^(μg N/l)	NH_4_^+^ (μg N/l)	(g/cm^3^)	TEX_86_	MBT	CBT	BIT	Cald/cren
**Lake water Depth(m)**												
												
0.5	12.00	12.57	7.39	−48.6	<5	<5	1.00201	0.28	0.34	1.39	0.81	1.32
3	11.75	12.65	7.40	−48.3	<5	<5	1.00201	0.27	0.32	1.28	0.74	1.21
6.6	11.70	12.35	7.38	−48.4	14	<5	1.00202	0.26	0.31	1.24	0.76	1.21
10.5	11.61	9.61	6.67	−47.3	9	<5	1.00305	0.31	0.30	0.93	0.32	1.20
14	11.15	10.06	6.78	−46.5	7	<5	1.00356	0.35	0.29	0.81	0.26	1.43
17	8.13	7.50	6.46	−46.1	12	<5	1.00438	0.32	0.28	0.92	0.20	1.32
19.5	4.04	0.57	6.15	−45.9	29	<5	1.00497	0.32	0.30	1.17	0.09	1.42
28	3.54	0.14	6.50	−42.8	6	1175	1.00673	0.34	0.27	1.32	0.20	1.72
44	3.41	0.14	6.54	−42.9	<5	3087	1.00683	0.33	0.34	1.03	0.39	1.58
**Surface sediment**												
Core top								0.36	0.36	1.42	0.28	1.4
												
**Stream water**												
Vendal					45	46		0.47	0.31	1.66	0.99	5.88
Lauvdal					45	<5		0.43	0.34	1.7	0.99	4.08
**Soil/peat samples**												
Soil beneath moss									0.52	1.50	1	
Lauvdal surface peat									0.45	1.46	1	
Lauvdal 1 m deep peat									0.49	1.51	1	
