# Supplementary material for: GDGT distribution in a stratified lake and implications for the application of TEX86 in paleoenvironmental reconstructions
Source: Sci Rep. 2016 Oct 3;6:34465. doi: 10.1038/srep34465 (PMC5046090; doi:10.1038/srep34465)
Supplement: Supplementary Information [file srep34465-s1.pdf]

GDGT distribution in a stratified lake and implications for the application of TEX_{86} in paleoenvironmental reconstructions

Zhaohui Zhang[1,2]*, Rienk H. Smittenberg[3] and Raymond S. Bradley[2]

1. Institute of Marine Chemistry and Environment, Ocean College, Zhejiang University, 1 Zheda Road, Zhoushan, 316021, China
2. Climate System Research Center, Department of Geosciences, University of Massachusetts, 627 North Pleasant Street, Amherst, MA 01003-9354, USA
3. Department of Geological Sciences, Stockholm University, Svante Arrhenius väg 8, SE-106 91 Stockholm, Sweden

*Corresponding author: Tel: +86-580-209-2264; email: zhaohui_zhang@zju.edu.cn

Supplementary Information

isoprenoid GDGTs

GDGT-I

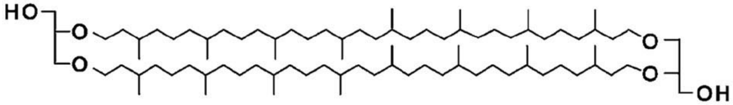

caldarchaeol

GDGT- II

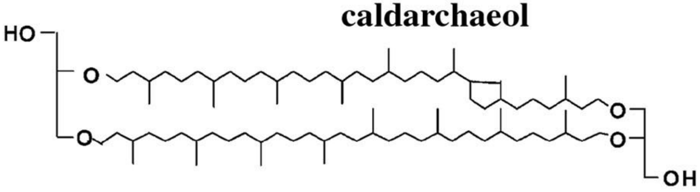

GDGT-III

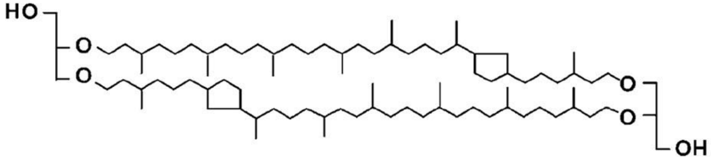

GDGT-IV

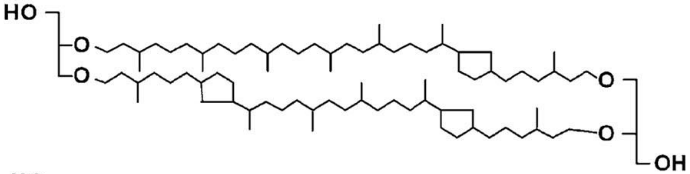

V(+V')
crenarchaeol
(+regio-isomer)

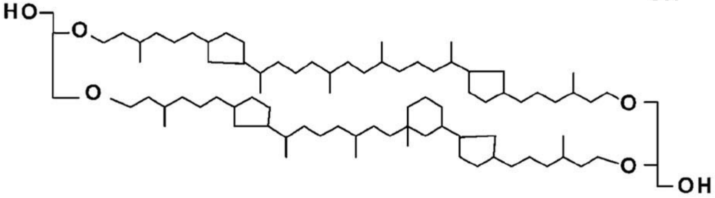

branched GDGTs

VI

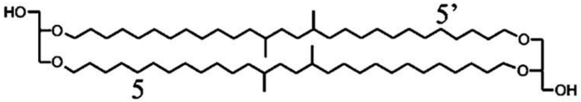

VIb

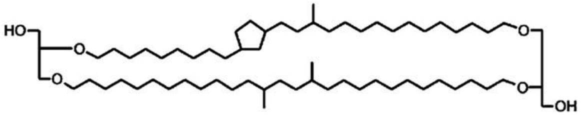

VIc

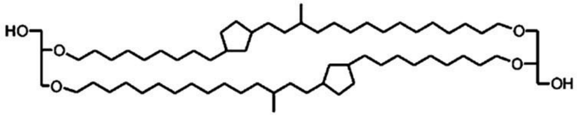

VII

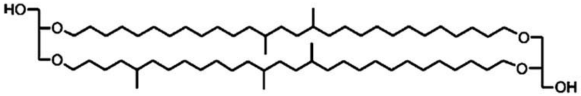

VIIb

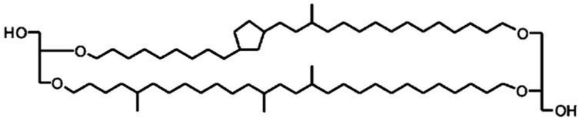

VIIc

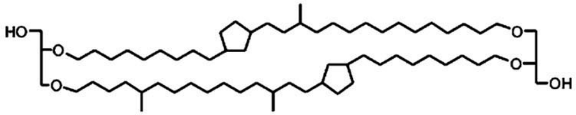

VIII

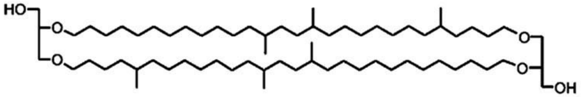

VIIIb

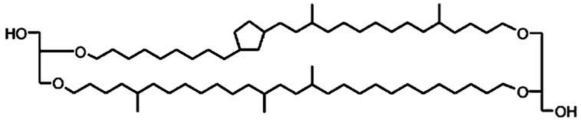

VIIIc

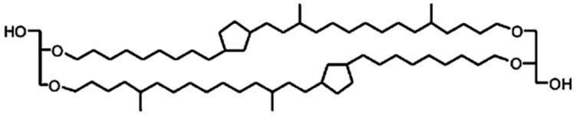

Table S1: Abundances of individual GDGTs in the water column of the lake, stream water flowing into the lake and surrounding soils

	Isoprenoid GDGTs						Branched GDGTs (ng g^{-1})									Σ(minor iso-GDGTs)	Σ(br-GDGTs)
	GDGT-I	GDGT-II	GDGT-III	GDGT-IV	GDGT-V	GDGT-V'	VI	VIb	VIc	VII	VIIb	VIIc	VIII	VIIIb	VIIIc		
**Lake water (ng l^{-1})**																	
Depth (m)																	
0.5	0.64	0.06	0.02	0	0.48	0	0.67	0.04	0	0.92	0.03	0	0.40	0.04	0.00	0.08	2.10
3.0	1.80	0.16	0.05	0	1.49	0.01	1.54	0.11	0	1.84	0.07	0	0.82	0.49	0.23	0.22	5.11
6.6	1.33	0.12	0.04	0	1.10	0.01	1.22	0.10	0	1.62	0.06	0	0.62	0.40	0.18	0.17	4.20
10.5	6.39	0.38	0.09	0.03	5.31	0.04	0.86	0.09	0	1.09	0.15	0	0.58	0.26	0.10	0.54	3.12
14.0	7.75	0.32	0.09	0.04	5.41	0.04	0.60	0.07	0	0.83	0.15	0	0.44	0.16	0.05	0.49	2.29
17.0	10.23	0.54	0.13	0.06	7.72	0.07	0.72	0.15	0	0.81	0.04	0	0.45	0.18	0.08	0.80	2.43
19.5	23.74	1.59	0.44	0.18	16.67	0.14	0.55	0.05	0	0.71	0.04	0	0.41	0.17	0.06	2.35	1.98
28.0	6.27	0.32	0.10	0.04	3.64	0.02	0.24	0.01	0	0.36	0.02	0	0.29	0	0	0.49	0.91
44.0	5.52	0.32	0.10	0.04	3.50	0.02	0.72	0.07	0.03	0.95	0.09	0	0.58	0	0	0.48	2.44
**Stream water (ng l^{-1})**																	
Vendal	1.04	0.16	0.13	0.01	0.18	0	5.33	0.29	0.10	7.70	0.24	0.12	4.36	0	0	0.14	18.14
Lauvdal	0.39	0.06	0.04	0	0.10	0	2.85	0.13	0.06	3.47	0.12	0.08	2.27	0	0	0.04	8.98
**Soil/peat samples (ng g^{-1} dried weight)**																	
Soil beneath moss	33.42	21.28	22.35	0	0	0	1776.37	77.76	13.12	1357.88	21.49	18.47	340.81	0	0	43.63	3605.89
Lauvdal surface peat	553.17	49.99	39.52	0	0	0	7896.22	459.67	92.16	7142.54	0	100.68	1715.54	0	0	89.51	17406.81
Lauvdal 1 m deep peat	2630.76	167.92	348.85	0	0	0	25887.86	1776.12	332.86	25280.93	0	396.42	8152.36	0	0	516.77	61826.55
**Core top[a]**																	
Core top																	

Note: a) Internal standard was missed during the core top sample processing. As a result abundances of individual GDGTs are not available.
